# Nonlinear association between stress hyperglycemia ratio and short-term and long-term mortality in sepsis patients with atrial fibrillation: a cohort study

**DOI:** 10.3389/fendo.2026.1920813

**Published:** 2026-08-26

**Authors:** Xiaomin Liang, Ying Li, Shuiqing Gui

**Affiliations:** Department of Critical Care Medicine, Shenzhen Second People’s Hospital, The First Affiliated Hospital of Shenzhen University, Shenzhen, Guangdong, China

**Keywords:** atrial fibrillation, mortality, nonlinear association, sepsis, stress hyperglycemia ratio

## Abstract

**Objective:**

The stress hyperglycemia ratio (SHR) demonstrates mortality associations across various populations; however, its nonlinear relationship with mortality in the specific subgroup of sepsis with atrial fibrillation (AF) remains insufficiently characterized.

**Methods:**

Retrospective cohort of sepsis patients with AF from Medical Information Mart for Intensive Care (MIMIC) -IV version 3.1 database. SHR was calculated as the ratio of admission glucose to estimated average glucose derived from hemoglobin A1c (HbA1c). Nonlinear associations between SHR and 30-day/365-day mortality were assessed using restricted cubic spline (RCS) and threshold effect analyses with multivariable adjustment and entropy balancing weighting.

**Results:**

Among 2,297 participants (median age 73.64 years, 63.2% male), 337 (14.67%) and 629 (27.38%) experienced 30-day and 365-day mortality. Nonlinear associations were identified between SHR and mortality, with inflection points at 0.70 (30-day) and 0.89 (365-day). Below thresholds, SHR was inversely associated with 30-day (HR = 0.10, 95% CI: 0.02–0.47, *P* = 0.003) and 365-day mortality (HR = 0.40, 95% CI: 0.20–0.78, *P* = 0.008). Above thresholds, mortality risk increased (30-day HR = 3.24, 95% CI: 2.19–4.80, *P* < 0.001; 365-day HR = 2.60, 95% CI: 1.80–3.76, *P* < 0.001). Entropy balancing weighting confirmed these threshold effects.

**Conclusion:**

A nonlinear relationship exists between SHR and mortality in sepsis patients with AF, where both extremely low and high SHR values may increase mortality risk.

## 1 Introduction

Sepsis represents a critical medical emergency characterized by dysregulated host immune responses to infectious agents, constituting a major burden on healthcare systems globally (1). The incidence and associated morbidity continue to escalate, creating substantial challenges for intensive care units (ICUs) and medical institutions worldwide (2, 3). This condition carries devastating short-term and long-term mortality outcomes, with 30-day fatality rates ranging from 20-40% and one-year mortality extending to 40-60% (4–8).

Atrial fibrillation (AF) is the most prevalent cardiac arrhythmia globally, affecting millions and substantially increasing morbidity and mortality through heart failure, hemodynamic compromise, and thromboembolic complications (9, 10). AF emerges as the predominant cardiac dysrhythmia in critically ill septic patients, with an incidence of 6-23%, driven by systemic inflammation, autonomic dysfunction, elevated circulating stress hormones, and intravascular volume fluctuations (11–16). When sepsis is complicated by AF, patients experience immediate hemodynamic deterioration, extended ICU length of stay, escalated healthcare expenditures, and markedly increased short-term and long-term risks of thromboembolism, cardiac failure, and mortality (17–20). Consequently, recognizing and managing risk factors represents a critical strategy for improving survival outcomes in patients with sepsis and AF.

Stress hyperglycemia, characterized by acute elevation of blood glucose concentrations during critical illness, frequently occurs in intensive care patients and correlates with poor clinical outcomes (21, 22). Since admission glucose values alone inadequately represent long-term glycemic status, hemoglobin A1c (HbA1c) serves as a valuable complement, reflecting mean glucose concentrations over the preceding 8–12 weeks to provide comprehensive glucose assessment (23). The stress hyperglycemia ratio (SHR) integrates both acute glucose levels and chronic glycemic control by incorporating admission glucose and HbA1c measurements (24). Multiple studies have demonstrated significant associations between elevated SHR and unfavorable patient outcomes (25–29).

However, its association with mortality in the specific subgroup of sepsis patients with AF remains insufficiently characterized. This study aimed to investigate the association between admission SHR and 30-day and 365-day mortality in critically ill sepsis patients with AF.

## 2 Methods

### 2.1 Data source and study population

This retrospective cohort study utilized the publicly available Medical Information Mart for Intensive Care-IV (MIMIC-IV) database version 3.1 (https://physionet.org/content/mimiciv/3.1/). The database contains clinical data from over 50,000 ICU admissions at Beth Israel Deaconess Medical Center, Boston, spanning 2008-2022 (30). Institutional review boards at Massachusetts Institute of Technology and Beth Israel Deaconess Medical Center provided ethical approval. Database access was obtained after completing the Collaborative Institutional Training Initiative certification (ID: 63069214). Since MIMIC-IV v3.1 contains completely de-identified patient information, informed consent was waived by the ethics committee (30). The study followed Declaration of Helsinki principles and STROBE (Strengthening the Reporting of Observational Studies in Epidemiology) reporting standards (31).

We collected data from patients with sepsis and AF. Sepsis was defined according to Sepsis 3.0 criteria (1): (1) suspected or confirmed infection, and (2) SOFA score ≥2. Patients with AF were identified using International Classification of Diseases, Ninth and Tenth Revision (ICD-9/10) codes (ICD-9: 427.31; ICD-10: I48.0, I48.1, I48.2, and I48.91) and categorized into two distinct subtypes: New-onset AF: AF occurring after ICU admission in patients without a documented prior AF history, confirmed by continuous bedside electrocardiographic monitoring and identified through heart rate status and rhythm entries in nursing flowsheets and clinical notes. Pre-existing AF: AF documented prior to ICU admission.

**Figure 1:**
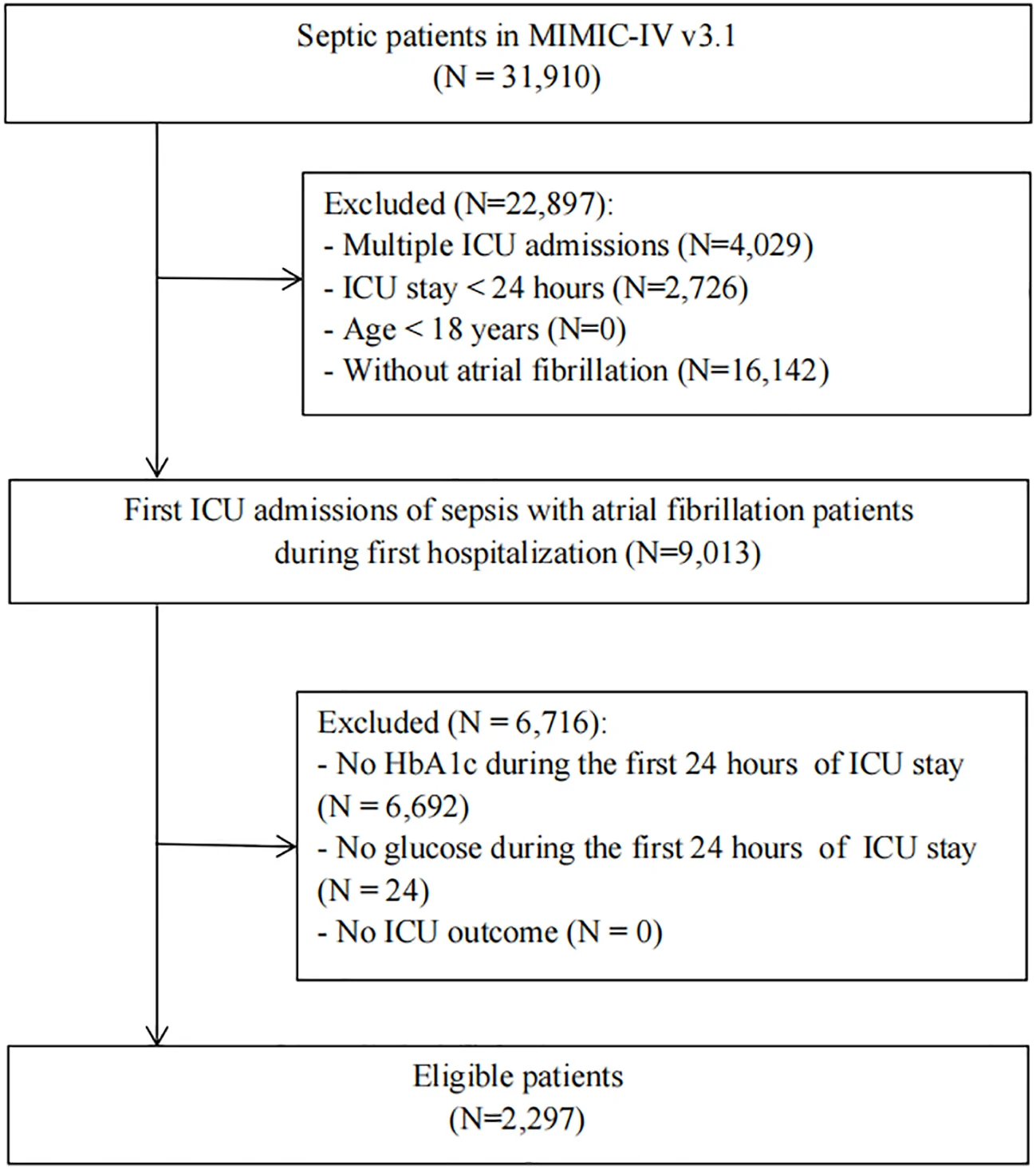
Flowchart of patient selection.

Exclusion criteria were: age < 18 years, multiple ICU admissions (non-first ICU stays), ICU stay < 24 hours, missing HbA1c or glucose values within the first 24 hours of ICU admission, and unavailable ICU outcome data. The final cohort comprised 2,297 sepsis patients with AF during their first ICU admission, as illustrated in **Figure 1**.

### 2.2 Data extraction

Clinical data were retrieved from the MIMIC-IV v3.1 database utilizing Navicat Premium (version 16.3.2). The following variables were collected: Demographic data: age, gender, race, and weight; First-day laboratory parameters: white blood cell (WBC), hemoglobin, platelet, blood urea nitrogen (BUN), creatinine, potassium, sodium, HbA1c, admission glucose, and international normalized ratio (INR); Comorbidity: myocardial infarction, congestive heart failure, peripheral vascular disease, cerebrovascular disease, chronic pulmonary disease, renal disease, diabetes, cancer, and AF type; Infection source and Sequential Organ Failure Assessment (SOFA) score; First-day interventions: mechanical ventilation, vasoactive agents, and continuous renal replacement therapy (CRRT).

### 2.3 Exposure and outcomes

#### 2.3.1 SHR calculation

HR was calculated using the formula originally proposed by Roberts et al. (24).


$$SHR=\frac{admission\;glucose\;(mg/dL)}{28.7\times HbA1c\;(\%)-46.7}$$

The numerator of SHR is the first glucose level (mg/dL) recorded within 24 hours of ICU admission. The denominator is the estimated average glucose (eAG, mg/dL) derived from HbA1c (%) using a validated formula from large population-based studies (24). To assess the temporal timing of baseline sampling, the exact time interval between ICU admission and the first blood glucose test was recorded.

#### 2.3.2 Outcomes and follow-up

The outcomes included 30-day and 365-day all-cause mortality, with follow-up duration calculated from ICU admission (defined as t=0). Vital status within 30 and 365 days post-ICU admission was determined by linking inpatient medical records with out-of-hospital death records embedded in MIMIC-IV (the dod field in the patients table).

### 2.4 Missing data

Missing data were observed for several variables: Weight (76, 3.31%), WBC (2, 0.09%), Hemoglobin (1, 0.04%), Platelet (2, 0.09%), and INR (37, 1.61%). Multiple imputation methods (32) were employed to handle incomplete values for these variables.

### 2.5 Statistical analysis

Data are presented as mean (SD), median [IQR], or n (%). Between-group comparisons used one-way ANOVA or Kruskal-Wallis test for continuous variables and chi-square test for categorical variables, as appropriate. The proportional hazards assumption for all Cox proportional hazards models was formally evaluated using scaled Schoenfeld residuals. Cox proportional hazards regression examined associations between SHR (continuous and quartiles, with Q1 as the reference) and 30-day and 365-day mortality, reported as hazard ratios (HRs) with 95% confidence intervals (CIs). A total of 26 covariates were selected based on prior literature (10, 11, 13, 17–20, 27, 28) and clinical relevance to outcomes, comprising gender, age, race, weight, myocardial infarction, congestive heart failure, peripheral vascular disease, cerebrovascular disease, chronic pulmonary disease, renal disease, diabetes, cancer, WBC, hemoglobin, platelet, BUN, creatinine, potassium, sodium, INR, SOFA, mechanical ventilation, vasoactive agent use, CRRT, AF type, and infection source.

To construct pseudo-populations and balance baseline confounders across SHR quartiles, entropy balancing weighting was performed using the WeightIt package (method = “ebal”, targeting the average treatment effect) in R. Entropy balancing directly optimizes sample weights to satisfy exact pre-specified balance constraints on baseline covariate moments (means), thereby eliminating confounding without severe weight instabilities (33). The weighting model incorporated 26 covariates. To further mitigate variance inflation caused by potential extreme weights, weights were Winsorized/truncated at the 0.1st and 99.9th percentiles. Weight distribution properties (mean, standard deviation, min, and max) and extreme weight diagnostics were evaluated. The weighted effective sample size (ESS) was calculated as $ESS={\left(\Sigma w_{\text{i}}\right)}^{2}/\Sigma w_{\text{i}}^{2}$ to quantify design efficiency (33). Covariate balance before and after weighting was assessed using Absolute Standardized Mean Differences (SMDs), with an SMD<0.10 predefined as successful balance.

To explore potential nonlinear associations between SHR and mortality, restricted cubic spline (RCS) regression with 4 knots (5th, 35th, 65th, and 95th percentiles) was applied. Optimal inflection points were identified using a recursive algorithm with maximum likelihood estimation, and likelihood ratio testing was used to compare the fit of two-piece segmented models with linear models (34). Threshold effect and RCS analyses were conducted with two approaches: multivariable adjustment alone, and with additional entropy balancing weighting as a sensitivity analysis to assess the robustness of findings. Prespecified subgroup analyses were performed across clinical strata, with interaction terms evaluated using the likelihood ratio test.

### 2.6 Sensitivity analyses

To evaluate potential nonlinear relationships between SHR and mortality outcomes, restricted cubic spline (RCS) models were fitted. To ensure the robustness of the identified nonlinear associations and threshold effects, the following sensitivity analyses were performed: (1) varying knot specifications (using 3 and 5 knots in addition to the primary 4-knot model); (2) altering the reference point to the cohort median SHR; (3) trimming extreme tail observations below the 1st percentile and above the 99th percentile; and (4) performing 1,000 bootstrap resamples to calculate 95% CIs for the threshold. The number of patients and events above and below each threshold was also tabulated.

To test the robustness of our primary exposure definition against early ICU therapeutics, sensitivity analyses were conducted using alternative glucose parameters derived within the first 24 hours of ICU stay, including the minimum glucose, maximum glucose, and mean glucose.

To quantify the potential impact of unmeasured residual confounding, E-values were calculated for both multivariable-adjusted and entropy balancing weighted models (35).

All statistical analyses were performed in R version 4.5.3 using the WeightIt, cobalt, rms, and survival packages. Two-tailed *P* < 0.05 was considered statistically significant.

## 3 Results

### 3.1 Baseline characteristics

Baseline characteristics of the 2,297 included sepsis patients with AF across SHR quartiles are presented in **Table 1**. The overall cohort had a median age of 73.64 years (IQR: 65.69–81.05), and 63.2% were male. The median time from ICU admission to the first blood glucose measurement was 3.38 hours (IQR: 0.54–14.00 hours). Prior to weighting, significant imbalances existed across quartiles in confounders, including diabetes, WBC, BUN, potassium, creatinine, and renal disease. Following entropy balancing across all covariates, covariate distributions were fully aligned (**Supplementary Figure 1**). Post-weighting SMDs for all covariates decreased from up to 0.288 to 0.012, far below the standard threshold of 0.10 (**Table 1**). The mean weight across all quartiles converged precisely to 1.00, with a maximum weight of 2.83 (**Supplementary Table 1**). The overall effective sample size (ESS) was 1,980.40 with an overall sample efficiency of 86.22% (**Supplementary Table 1**).

**Table 1:** Baseline characteristics of participants across SHR quartiles before and after entropy balancing weighting.

Variable	Overall (n=2,297)	Q1 (0.19–0.78) (n=575)	Q2 (0.78–0.93) (n=574)	Q3 (0.93–1.11) (n=574)	Q4 (1.11–2.55) (n=574)	Unweighted SMD	Weighted SMD
Demographics							
Male, n (%)	1,451 (63.2)	361 (62.8)	370 (64.5)	376 (65.5)	344 (59.9)	0.056	< 0.001
Age, years, median [IQR]	73.64 [65.69, 81.05]	73.59 [65.30, 81.57]	74.26 [66.30, 80.66]	73.67 [66.26, 80.77]	73.11 [65.01, 81.08]	0.048	< 0.001
White, n (%)	1,551 (67.5)	384 (66.8)	384 (66.9)	405 (70.6)	378 (65.9)	0.047	< 0.001
Weight, kg, median [IQR]	82.40 [69.40, 98.30]	82.00 [69.00, 98.20]	82.70 [69.40, 98.22]	82.35 [69.53, 98.07]	82.20 [70.00, 99.92]	0.074	< 0.001
Comorbidities							
Myocardial infarction, n (%)	721 (31.4)	192 (33.4)	168 (29.3)	171 (29.8)	190 (33.1)	0.041	< 0.001
Congestive heart failure, n (%)	1,117 (48.6)	287 (49.9)	289 (50.3)	260 (45.3)	281 (49.0)	0.051	< 0.001
Peripheral vascular disease, n (%)	398 (17.3)	91 (15.8)	102 (17.8)	106 (18.5)	99 (17.2)	0.026	< 0.001
Cerebrovascular disease, n (%)	637 (27.7)	145 (25.2)	151 (26.3)	178 (31.0)	163 (28.4)	0.058	< 0.001
Chronic pulmonary disease, n (%)	610 (26.6)	159 (27.7)	156 (27.2)	152 (26.5)	143 (24.9)	0.027	< 0.001
Renal disease, n (%)	649 (28.3)	207 (36.0)	151 (26.3)	136 (23.7)	155 (27.0)	0.123	< 0.001
Diabetes, n (%)	672 (29.3)	273 (47.5)	151 (26.3)	114 (19.9)	134 (23.3)	0.276	< 0.001
Cancer, n (%)	142 (6.2)	35 (6.1)	34 (5.9)	37 (6.4)	36 (6.3)	0.005	< 0.001
Laboratory parameters							
WBC, ×10^9^/L, median [IQR]	14.60 [10.90, 18.70]	14.60 [10.70, 18.55]	13.75 [10.10, 18.20]	14.50 [11.20, 18.10]	15.45 [12.12, 19.70]	0.279	0.012
Hemoglobin, g/dL, median [IQR]	9.50 [8.10, 11.20]	9.30 [8.10, 11.00]	9.50 [8.00, 11.17]	9.80 [8.40, 11.50]	9.60 [8.10, 11.28]	0.195	< 0.001
Platelet, ×10^9^/L, median [IQR]	146.00 [109.00, 200.00]	147.00 [111.00, 198.00]	143.50 [109.00, 197.00]	146.00 [108.25, 198.75]	147.00 [105.00, 205.00]	0.068	< 0.001
BUN, mg/dL, median [IQR]	23.00 [16.00, 36.00]	25.00 [17.00, 42.00]	21.00 [16.00, 32.00]	21.00 [15.00, 31.00]	24.00 [16.00, 39.75]	0.260	< 0.001
Creatinine, mg/dL, median [IQR]	1.20 [0.90, 1.70]	1.20 [0.90, 2.00]	1.10 [0.90, 1.50]	1.10 [0.80, 1.60]	1.20 [0.90, 1.70]	0.210	< 0.001
Potassium, mmol/L, median [IQR]	4.60 [4.20, 5.00]	4.70 [4.30, 5.20]	4.60 [4.30, 4.90]	4.50 [4.20, 4.90]	4.50 [4.20, 4.90]	0.288	< 0.001
Sodium, mmol/L, median [IQR]	140.00 [137.00, 142.00]	140.00 [137.00, 142.00]	140.00 [137.25, 142.00]	139.00 [137.00, 142.00]	139.00 [137.00, 142.00]	0.164	< 0.001
INR, median [IQR]	1.40 [1.30, 1.70]	1.40 [1.30, 1.70]	1.40 [1.30, 1.70]	1.40 [1.20, 1.70]	1.40 [1.20, 1.60]	0.073	< 0.001
Clinical interventions							
Mechanical ventilation, n (%)	1,512 (65.8)	372 (64.7)	388 (67.6)	378 (65.9)	374 (65.2)	0.029	< 0.001
Vasoactive agent use, n (%)	1,234 (53.7)	336 (58.4)	316 (55.1)	296 (51.6)	286 (49.8)	0.086	< 0.001
CRRT, n (%)	97 (4.2)	35 (6.1)	16 (2.8)	19 (3.3)	27 (4.7)	0.033	< 0.001
AF and Sepsis characteristics							
AF type, n (%)							
New-onset	1,379 (60.0)	350 (60.9)	362 (63.1)	348 (60.6)	319 (55.6)	—	—
Pre-existing	918 (40.0)	225 (39.1)	212 (36.9)	226 (39.4)	255 (44.4)	0.075	< 0.001
Infection source, n (%)							
Respiratory	1,079 (47.0)	308 (53.6)	287 (50.0)	260 (45.3)	224 (39.0)	0.145	< 0.001
Urinary	439 (19.1)	86 (15.0)	106 (18.5)	117 (20.4)	130 (22.6)	0.077	< 0.001
Bloodstream	387 (16.8)	90 (15.7)	90 (15.7)	89 (15.5)	118 (20.6)	0.051	< 0.001
Other	392 (17.1)	91 (15.8)	91 (15.9)	108 (18.8)	102 (17.8)	0.030	< 0.001
SOFA score, median [IQR]	3.00 [2.00, 5.00]	3.00 [2.00, 5.00]	3.00 [2.00, 5.00]	3.00 [2.00, 5.00]	3.00 [2.00, 5.00]	0.056	< 0.001

Continuous variables are presented as median [interquartile range (IQR)]; categorical variables are presented as n (%). Covariate balance across SHR quartiles was evaluated using the Standardized Mean Difference (SMD) before and after entropy balancing weighting. SMD, standardized mean difference; SHR, stress hyperglycemia ratio; AF, atrial fibrillation; WBC, white blood cell; BUN, blood urea nitrogen; INR, international normalized ratio; SOFA, Sequential Organ Failure Assessment; CRRT, continuous renal replacement therapy.

The clinical outcomes of the unweighted cohort stratified by SHR quartiles are detailed in **Table 2**. In the overall population ( N=2,297), the 30-day and 365-day all-cause mortality rates were 14.67% ( n=337) and 27.38% ( n=629), respectively. Both short- and long-term mortality rates differed significantly across the four SHR quartiles. Specifically, patients in the highest quartile (Q4) experienced the highest mortality rates, whereas patients in the second quartile (Q2) exhibited the lowest mortality rates (**Table 2**).

**Table 2:** Clinical outcomes of patients stratified by SHR quartiles.

Outcome	Overall n=2,297	Q1 (0.19–0.78) n=575	Q2 (0.78–0.93) n=574	Q3 (0.93–1.11) n=574	Q4 (1.11–2.55) n=574	*P*
30-Day Mortality, n (%)	337 (14.67)	73 (12.70)	64 (11.15)	81 (14.11)	119 (20.73)	<0.001
365-Day Mortality, n (%)	629 (27.38)	156 (27.13)	140 (24.39)	145 (25.26)	188 (32.75)	0.007

Data are presented as number (percentage). *P* values were calculated using the Pearson Chi-squared test comparing the rates across the four SHR quartiles. SHR, stress hyperglycemia ratio; Q, quartile.

### 3.2 Association between SHR and mortality

Schoenfeld residual diagnostics indicated that the proportional hazards assumption was fully satisfied (global test: *P* = 0.603). When analyzed continuously, higher SHR was consistently associated with increased 30-day and 365-day mortality across all models (**Table 3**). In quartile analyses, Q2 showed numerically lower mortality risk than Q1, with risk increasing in Q3 and Q4. For 30-day mortality, the elevated risk in Q4 remained significant across unadjusted, multivariable-adjusted, and entropy-balancing weighted models. For 365-day mortality, Q4 reached statistical significance in unadjusted and multivariable-adjusted models, while showing a borderline trend in the weighted model (**Table 3**).

**Table 3:** Association between SHR and mortality.

Variable	Unadjusted		Multivariable-adjusted		Entropy balancing weighted	
	HR (95% CI)	*P*	HR (95% CI)	*P*	HR (95% CI)	*P*
30-Day Mortality						
SHR (continuous)	2.36 (1.67–3.32)	< 0.001	2.15 (1.50–3.08)	< 0.001	2.32 (1.49–3.61)	< 0.001
SHR quartiles						
Q1 (0.19–0.78)	Reference		Reference		Reference	
Q2 (0.78–0.93)	0.86 (0.62–1.21)	0.385	0.90 (0.64–1.27)	0.561	0.98 (0.69–1.38)	0.888
Q3 (0.93–1.11)	1.11 (0.81–1.53)	0.510	1.15 (0.83–1.60)	0.402	1.22 (0.86–1.71)	0.267
Q4 (1.11–2.55)	1.70 (1.27–2.28)	< 0.001	1.58 (1.17–2.14)	0.003	1.66 (1.19–2.31)	0.003
*P* for trend	< 0.001		< 0.001		0.001	
365-Day Mortality						
SHR (continuous)	1.54 (1.18–2.02)	0.002	1.49 (1.12–1.97)	0.006	1.55 (1.11–2.19)	0.011
SHR quartiles						
Q1 (0.19–0.78)	Reference		Reference		Reference	
Q2 (0.78–0.93)	0.88 (0.70–1.11)	0.271	0.91 (0.72–1.15)	0.443	0.96 (0.75–1.23)	0.742
Q3 (0.93–1.11)	0.93 (0.74–1.16)	0.511	0.95 (0.75–1.20)	0.683	1.01 (0.78–1.28)	0.992
Q4 (1.11–2.55)	1.28 (1.04–1.59)	0.021	1.25 (1.01–1.56)	0.041	1.26 (0.99–1.61)	0.058
*P* for trend	0.017		0.039		0.058	

Unadjusted: Unadjusted for any covariates. Multivariable-adjusted model was adjusted for gender, age, race, weight, myocardial infarction, congestive heart failure, peripheral vascular disease, cerebrovascular disease, chronic pulmonary disease, renal disease, diabetes, cancer, WBC, hemoglobin, platelet, BUN, creatinine, potassium, sodium, INR, SOFA score, mechanical ventilation, vasoactive agent use, CRRT, AF type, and infection source. Weighted Cox proportional hazards models were weighted using entropy balancing across all 26 baseline covariates to achieve optimal covariate balance. *P* for trend was calculated by treating the SHR quartiles as a continuous ordinal variable in the corresponding Cox models. SHR, stress hyperglycemia ratio; HR, hazard ratio; CI, confidence interval.

### 3.3 Nonlinear relationship between SHR and mortality

**Figure 2:**
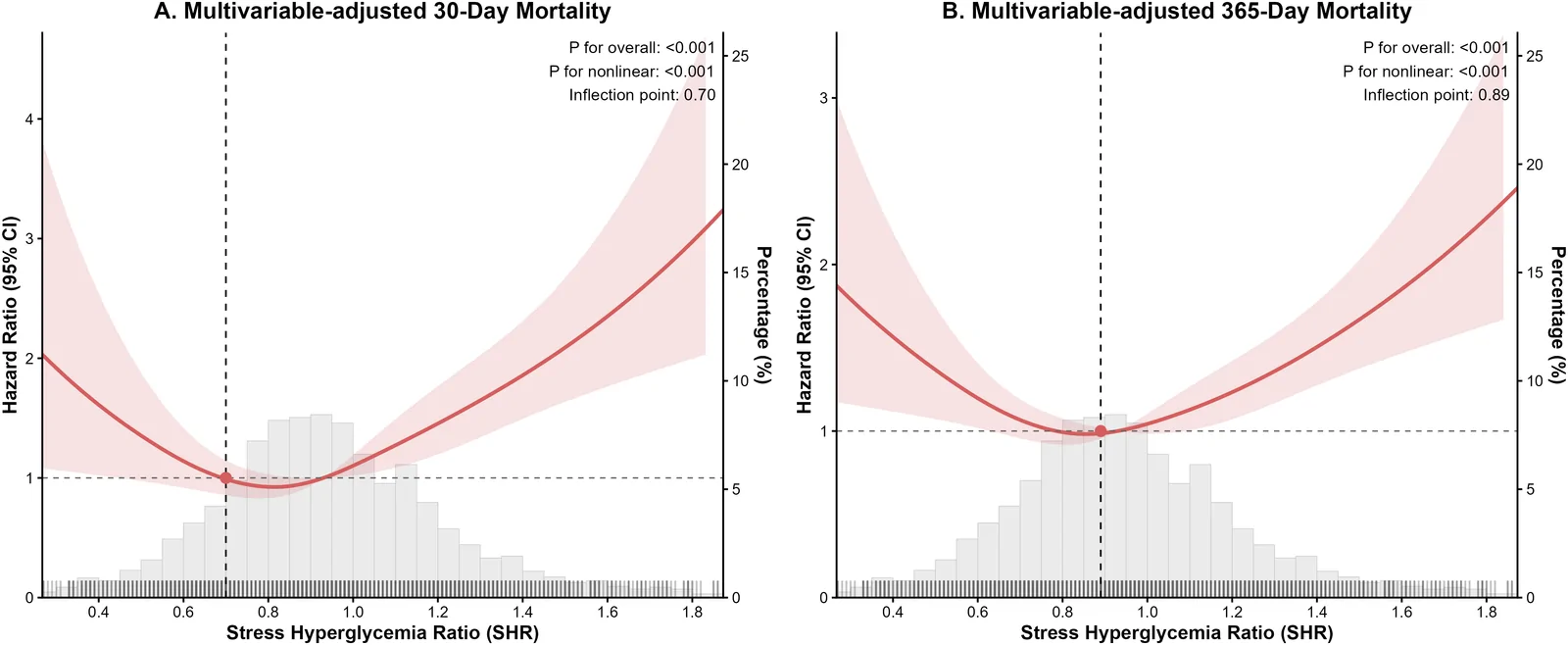
RCS analysis of SHR and mortality (multivariable-adjusted model). **(A)** 30-day mortality; **(B)** 365-day mortality. Dose-response relationship between SHR and 30-day **(A)** and 365-day **(B)** mortality. Red lines represent hazard ratios; shaded areas indicate 95% CIs. Inflection points were 0.70 for 30-day and 0.89 for 365-day mortality (both *P* for overall < 0.001, *P* for nonlinearity < 0.001). RCS models used 4 knots, adjusted for gender, age, race, weight, myocardial infarction, congestive heart failure, peripheral vascular disease, cerebrovascular disease, chronic pulmonary disease, renal disease, diabetes, cancer, WBC, hemoglobin, platelet, BUN, creatinine, potassium, sodium, INR, SOFA score, mechanical ventilation, vasoactive agent use, CRRT, AF type, and infection source. SHR, stress hyperglycemia ratio; RCS, restricted cubic spline; CI, confidence interval.

**Figure 3:**
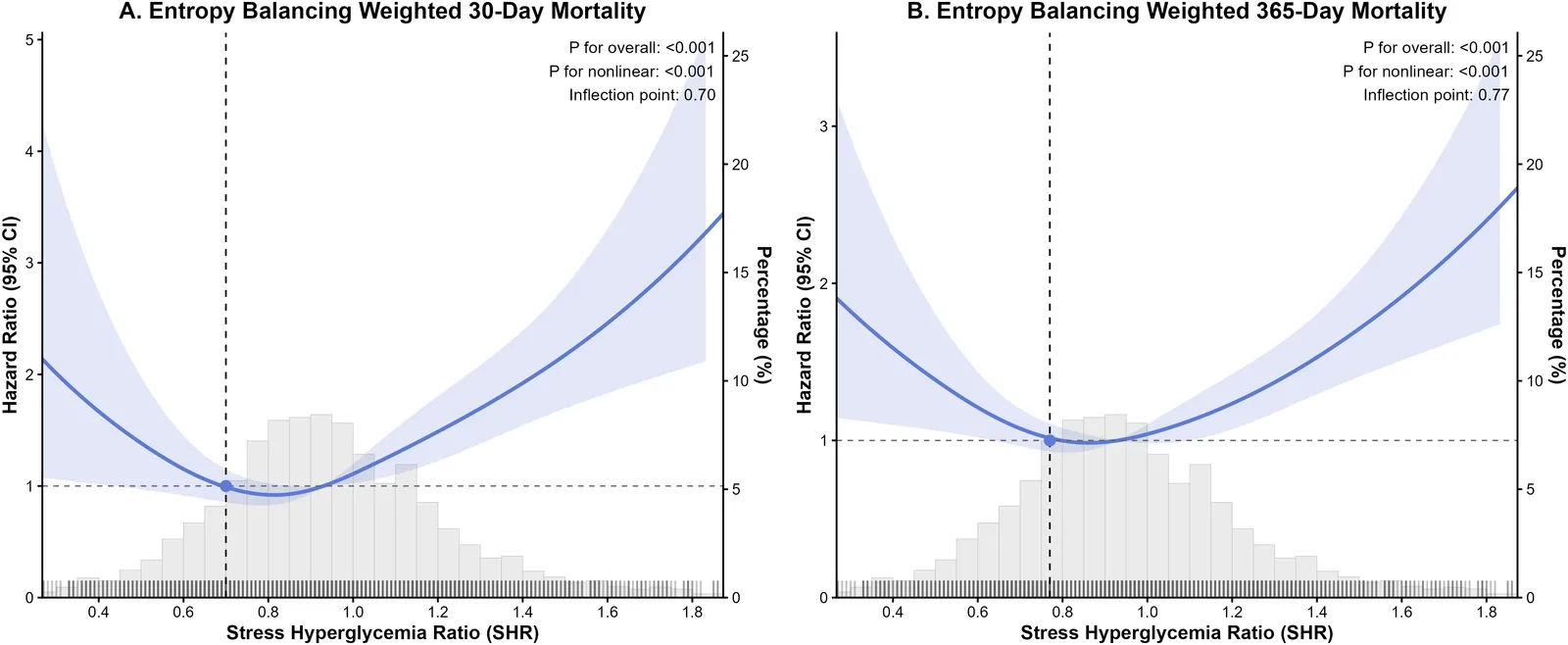
RCS analysis after entropy balancing weighting. **(A)** 30-day mortality; **(B)** 365-day mortality. Red lines represent hazard ratios; shaded areas indicate 95% CIs. Inflection points were 0.70 for 30-day and 0.77 for 365-day mortality (both *P* for overall < 0.001, *P* for nonlinearity < 0.001). SHR, stress hyperglycemia ratio; RCS, restricted cubic spline; CI, confidence interval.

RCS analysis revealed a significant nonlinear relationship between SHR and mortality in both the multivariable-adjusted and entropy balancing weighted analyses (**Figures 2**, **3**).

Two-piecewise Cox proportional hazards models identified nonlinear relationships between SHR and mortality (**Table 4**). For 30-day mortality, the inflection point was 0.70 (95% CI: 0.58–0.77). Below 0.70, SHR was inversely associated with mortality risk (multivariable-adjusted HR = 0.10, 95% CI: 0.02–0.47, *P* = 0.003; entropy balancing weighted HR = 0.07, 95% CI: 0.01–0.33, *P* = 0.001), whereas above 0.70, mortality risk increased (multivariable-adjusted HR = 3.24, 95% CI: 2.19–4.80, *P* < 0.001; weighted HR = 3.37, 95% CI: 2.28–4.98, *P* < 0.001). For 365-day mortality, inflection points were 0.89 (multivariable-adjusted, 95% CI: 0.83–0.94) and 0.77 (weighted, 95% CI: 0.68–0.82). Below these thresholds, SHR was negatively associated with mortality (multivariable-adjusted HR = 0.40, 95% CI: 0.20–0.78, *P* = 0.008; weighted HR = 0.19, 95% CI: 0.07–0.51, *P* = 0.001); above them, mortality risk elevated (multivariable-adjusted HR = 2.60, 95% CI: 1.80–3.76, *P* < 0.001; weighted HR = 2.32, 95% CI: 1.67–3.23, *P* < 0.001).

**Table 4:** Threshold effect analysis of SHR on mortality.

Outcome	Segment	N (events)	Multivariable-adjusted		Entropy balancing weighted	
			HR (95% CI)	*P*	HR (95% CI)	*P*
30-Day mortality	Inflection point (K, 95% CI)	—	0.70 (0.58- 0.77)	—	0.70 (0.58- 0.77)	—
	SHR ≤ K	372 (48)	0.10 (0.02-0.47)	0.003	0.07 (0.01–0.33)	0.001
	SHR > K	1925 (289)	3.24 (2.19-4.80)	<0.001	3.37 (2.28–4.98)	<0.001
365-Day mortality	Inflection point (K, 95% CI)	—	0.89 (0.83- 0.94)	—	0.77 (0.68- 0.82)	—
	SHR ≤ K	1004 (259)	0.40 (0.20-0.78)	0.008	0.19 (0.07–0.51)	0.001
	SHR > K	1293 (370)	2.60 (1.80-3.76)	<0.001	2.32 (1.67–3.23)	<0.001

N (events) represents the unweighted observed number of patients and mortality events in each segment. Multivariable-adjusted model was adjusted for gender, age, race, weight, myocardial infarction, congestive heart failure, peripheral vascular disease, cerebrovascular disease, chronic pulmonary disease, renal disease, diabetes, cancer, WBC, hemoglobin, platelet, BUN, creatinine, potassium, sodium, INR, SOFA score, mechanical ventilation, vasoactive agent use, CRRT, AF type, and infection source. Entropy Balancing Weighted models were weighted across all 26 covariates to target the average treatment effect. Inflection points were calculated using two-piecewise Cox proportional hazards models. HR, hazard ratio; CI, confidence interval; SHR, stress hyperglycemia ratio; K, inflection point (threshold).

### 3.4 Subgroup analyses

Elevated SHR demonstrated consistent associations with increased 30-day and 365-day mortality (HR 2.33 and 1.56, respectively). Subgroup analyses revealed homogeneous effects across gender, age, race, cerebrovascular disease, renal disease, diabetes, HbA1c, AF type, infection source, SOFA, and vasoactive agents use (**Supplementary Figure 2**).

### 3.5 Sensitivity analyses

Sensitivity analyses using different knot numbers (**Supplementary Figure 3**), different reference values (**Supplementary Figure 4**), trimming of extreme SHR values (**Supplementary Figure 5**) produced identical nonlinear curves.

Sensitivity analyses using alternative glucose parameters during the first 24 hours of ICU admission confirmed robust nonlinear threshold effects on 30-day mortality across admission, minimum, and mean SHR metrics (**Supplementary Table 3**). Consistent threshold patterns were likewise observed for 365-day mortality.

E-value quantitative evaluation confirmed robustness against unmeasured residual confounding (**Supplementary Table 4**).

## 4 Discussion

This retrospective cohort study using the MIMIC-IV v3.1 database suggests a nonlinear association between SHR and 30-day (short-term) and 365-day (long-term) mortality in sepsis patients with AF. Cox proportional hazards regression quantified the linear association, while multivariable-adjusted RCS and threshold effect analysis explored potential nonlinear relationships. The entropy balancing weighted sensitivity analysis yielded consistent findings, confirming the robustness of this nonlinear association.

This study identified a nonlinear relationship between SHR and mortality in sepsis patients with AF, with inflection points at 0.70 (30-day) and 0.89 (365-day). Compared with Li et al. (27) (inflection point 0.99 in sepsis cohort), the present findings show lower thresholds. However, consistency was observed with Cheng et al.’s AF cohort (28) (inflection points 0.73 and 0.76). The present study extends prior research to the specific subgroup with concurrent sepsis and AF. These findings suggest that the relationship between glycemic dysregulation and mortality differs between sepsis patients with AF and those with sepsis alone, a hypothesis that warrants further investigation in prospective studies.

This study demonstrates both extremely low and high SHR values increase mortality risk, involving two opposing pathophysiological mechanisms. Low SHR reflects relative hypoglycemia, indicating severe metabolic dysfunction and hepatic glycogen depletion (21, 22, 36). Under dual stress of AF and sepsis, increased metabolic demands with insufficient glycogen reserves cause cellular energy imbalance, particularly affecting cardiac and cerebral metabolism, thereby increasing mortality (15, 16). High SHR reflects excessive stress hyperglycemia, causing endothelial dysfunction, enhanced inflammation, coagulation abnormalities, and immunosuppression (21, 37, 38). In AF patients, hyperglycemia may worsen arrhythmias, hemodynamic instability, and thrombotic risk (9, 10, 39). The mismatch between chronic glycemic control and acute glucose elevation reflects maladaptive stress response and limited physiological reserve (24). The inflection points for short-term and long-term mortality identified in this study represent exploratory findings. These findings suggest that both insufficient glycemic control and excessive stress-induced hyperglycemia may be associated with increased mortality risk, highlighting the potential importance of individualized glycemic monitoring in this population. Prospective studies and external validation are warranted to confirm whether these thresholds hold clinical utility.

This study has several strengths. First, this study extends the current literature by specifically characterizing the nonlinear association between SHR and both short-term and long-term mortality in the high-risk subgroup of sepsis patients with concurrent AF. Second, the large sample size of 2,297patients from MIMIC-IV database, combined with advanced statistical methodologies including multivariable-adjusted RCS and threshold effect analysis explored potential nonlinear relationships. Third, to account for potential baseline covariate imbalance across SHR quartiles, we performed entropy balancing weighting as a sensitivity analysis, which confirmed the robustness of our primary findings. Several limitations warrant consideration. First, the retrospective observational design inherently precludes causal inference. Although we adjusted for multiple confounders, balanced 26 baseline covariates thorough entropy balancing weighting, and demonstrated robust E-values against unmeasured confounding, residual confounding from unmeasured factors cannot be completely ruled out. Second, the inflection points identified were exploratory findings from a single dataset. The wide CIs of subthreshold HRs (due to sparse events) limit the precision of effect estimates in this range. Although consistent with the nonlinear pattern, the clinical significance is limited. Third, 74.2% of patients were excluded due to unavailable HbA1c within the first 24 hours of ICU admission, introducing potential selection bias (**Supplementary Table 2**). Our findings primarily apply to critically ill patients undergoing early metabolic evaluation rather than an unselected general ICU population. Fourth, this study used admission SHR values and did not capture temporal changes in glucose levels during ICU stay. Future investigations incorporating serial glucose measurements or time-varying glycemic indices are needed to characterize the dynamic relationship between glycemic control and outcomes.

## 5 Conclusion

Among 2,297 sepsis patients with AF from MIMIC-IV v3.1, we identified a nonlinear relationship between SHR and mortality. Very low SHR and high SHR may both be associated with increased mortality risk. Given the single-center derivation and lack of external validation, these findings should be considered exploratory and require prospective validation before clinical implementation.

## Data Availability

The original contributions presented in the study are included in the article/**Supplementary Material**. Further inquiries can be directed to the corresponding authors.
