# Supplementary material for: Nonlinear association between stress hyperglycemia ratio and short-term and long-term mortality in sepsis patients with atrial fibrillation: a cohort study
Source: Front Endocrinol (Lausanne). 2026 Aug 26;17:1920813. doi: 10.3389/fendo.2026.1920813 (PMC13553279; doi:10.3389/fendo.2026.1920813)
Supplement: Supplementary file 1 [file Supplementaryfile1.docx]

Supplementary Material

**Table S1. Summary of entropy balancing weight distributions and effective sample sizes across SHR quartiles**

| **Parameter / Diagnostic Metric** | **Quartile 1 (Q1)** | **Quartile 2 (Q2)** | **Quartile 3 (Q3)** | **Quartile 4 (Q4)** | **Overall Cohort** |
| --- | --- | --- | --- | --- | --- |
| **Raw Sample Size (N)** | 575 | 574 | 574 | 574 | 2,297 |
| **Weight Distribution** | | | | | |
| Mean ± SD | 1.00 ± 0.54 | 1.00 ± 0.21 | 1.00 ± 0.40 | 1.00 ± 0.39 | 1.00 ± 0.40 |
| Median (IQR) | 0.90 (0.57-1.34) | 0.98 (0.86-1.10) | 0.91 (0.73-1.19) | 0.94 (0.74-1.21) | 0.94 (0.75-1.18) |
| Minimum | 0.12 | 0.60 | 0.42 | 0.12 | 0.12 |
| Maximum  (Truncated at 99.9%) | 2.83 | 2.42 | 2.83 | 2.75 | 2.83 |
| **Effective Sample Size Diagnostics** | | | | | |
| Effective Sample Size (ESS) | 445.94 | 550.74 | 495.52 | 499.43 | 1,980.40 |
| Sample Efficiency (%) | 77.55% | 95.95% | 86.33% | 87.01% | 86.22% |
| Efficiency Loss (%) | 22.45% | 4.05% | 13.67% | 12.99% | 13.78% |
| **Covariate Balance Metric** | | | | | |
| Maximum Post-weighting SMD | <0.012 | <0.012 | <0.012 | <0.012 | <0.012 |

Effective Sample Size (ESS) was calculated using the formula $ESS = (\Sigma wᵢ)^{2} / \Sigma wᵢ^{2}.$ Weights were derived using entropy balancing (method = "ebal", targeting ATE) and Winsorized/truncated at the 0.1st and 99.9th percentiles. Abbreviations: SHR, Stress Hyperglycemia Ratio; SD, Standard Deviation; IQR, Interquartile Range; ESS, Effective Sample Size; SMD, Standardized Mean Difference.

**Table S2. Comparison of baseline characteristics and outcomes between included and excluded patients**

| **Variable** | **Included**  **(N = 2,297)** | **Excluded**  **(N = 6,692)** | **SMD (95% CI)** | ***P*** |
| --- | --- | --- | --- | --- |
| **Demographics** |  |  |  |  |
| Male, n (%) | 1,451 (63.17%) | 3,939 (58.86%) | 0.09 (0.04, 0.14) | <0.001 |
| Age, years, median [IQR] | 73.64 [65.69–81.05] | 76.07 [66.82–83.92] | 0.17 (0.12, 0.21) | <0.001 |
| White, n (%) | 1,551 (67.52%) | 4,861 (72.64%) | 0.11 (0.06, 0.16) | <0.001 |
| Weight, kg, median [IQR] | 82.20 [69.25–98.15] | 80.00 [66.90–95.00] | 0.10 (0.05, 0.15) | <0.001 |
| **Comorbidities** |  |  |  |  |
| Myocardial infarction, n (%) | 721 (31.39%) | 1,314 (19.64%) | 0.27 (0.22, 0.32) | <0.001 |
| Congestive heart failure, n (%) | 1,117 (48.63%) | 3,017 (45.08%) | 0.07 (0.02, 0.12) | 0.003 |
| Peripheral vascular disease, n (%) | 398 (17.33%) | 1,057 (15.79%) | 0.04 (-0.01, 0.09) | 0.085 |
| Cerebrovascular disease, n (%) | 637 (27.73%) | 844 (12.61%) | 0.38 (0.34, 0.43) | <0.001 |
| Chronic pulmonary disease, n (%) | 610 (26.56%) | 2,052 (30.66%) | 0.09 (0.04, 0.14) | <0.001 |
| Renal disease, n (%) | 649 (28.25%) | 2,031 (30.35%) | 0.05 (-0.00, 0.09) | 0.058 |
| Diabetes, n (%) | 672 (29.26%) | 1,635 (24.43%) | 0.11 (0.06, 0.16) | <0.001 |
| Cancer, n (%) | 142 (6.18%) | 992 (14.82%) | 0.28 (0.24, 0.33) | <0.001 |
| **Laboratory Parameters** |  |  |  |  |
| WBC, ×10⁹/L, median [IQR] | 14.60 [10.90–18.70] | 14.00 [10.10–19.20] | 0.01 (-0.04, 0.06) | 0.008 |
| Hemoglobin, g/dL, median [IQR] | 9.50 [8.10–11.20] | 9.60 [8.20–11.10] | 0.04 (-0.01, 0.08) | 0.698 |
| Platelet, ×10⁹/L, median [IQR] | 146.00 [109.00–200.00] | 157.00 [110.00–220.00] | 0.14 (0.10, 0.19) | <0.001 |
| BUN, mg/dL, median [IQR] | 23.00 [16.00–36.00] | 28.00 [18.00–46.00] | 0.26 (0.21, 0.31) | <0.001 |
| Creatinine, mg/dL, median [IQR] | 1.20 [0.90–1.70] | 1.30 [0.90–2.10] | 0.16 (0.11, 0.20) | <0.001 |
| Potassium, mmol/L, median [IQR] | 4.60 [4.20–5.00] | 4.50 [4.10–5.00] | 0.02 (-0.03, 0.06) | 0.014 |
| Sodium, mmol/L, median [IQR] | 140.00 [137.00–142.00] | 140.00 [137.00–143.00] | 0.06 (0.01, 0.11) | <0.001 |
| INR, median [IQR] | 1.40 [1.30–1.70] | 1.40 [1.20–1.90] | 0.25 (0.20, 0.30) | <0.001 |
| **Clinical Interventions** |  |  |  |  |
| Mechanical ventilation, n (%) | 1,512 (65.82%) | 3,808 (56.90%) | 0.18 (0.14, 0.23) | <0.001 |
| Vasoactive agent use, n (%) | 1,234 (53.72%) | 3,259 (48.70%) | 0.10 (0.05, 0.15) | <0.001 |
| CRRT, n (%) | 97 (4.22%) | 412 (6.16%) | 0.09 (0.04, 0.13) | <0.001 |
| **AF and Sepsis Characteristics** |  |  |  |  |
| Pre-existing AF, n (%) | 918 (39.97%) | 2,130 (31.83%) | 0.17 (0.12, 0.22) | <0.001 |
| Respiratory infection, n (%) | 1,077 (46.89%) | 3,722 (55.62%) | 0.18 (0.13, 0.22) | <0.001 |
| SOFA score, median [IQR] | 3.00 [2.00–5.00] | 3.00 [2.00–5.00] | 0.04 (-0.01, 0.09) | 0.138 |
| **Outcomes** |  |  |  |  |
| 30-Day mortality, n (%) | 337 (14.67%) | 1,600 (23.91%) | 0.24 (0.19, 0.28) | <0.001 |
| 365-Day mortality, n (%) | 629 (27.38%) | 2,720 (40.65%) | 0.28 (0.24, 0.33) | <0.001 |

Abbreviations: IQR, interquartile range; SMD, standardized mean difference; WBC, white blood cell; BUN, blood urea nitrogen; INR, international normalized ratio; CRRT, continuous renal replacement therapy; AF, atrial fibrillation; SOFA, Sequential Organ Failure Assessment.

**Table S3. Sensitivity analysis of threshold effects on 30-day and 365-day mortality using different definitions of SHR**

| **Outcome / Model** | **Admission SHR** | **Minimum SHR** | **Maximum SHR** | **Mean SHR** |
| --- | --- | --- | --- | --- |
|  | **HR (95% CI)**   ***P*** | **HR (95% CI)**   ***P*** | **HR (95% CI)**   ***P*** | **HR (95% CI)**   ***P*** |
| **30-Day Mortality** | | | | |
| **Model 1: Linear Cox** | 2.24 (1.56-3.21)   <0.001 | 2.28 (1.56-3.32)   <0.001 | 1.25 (1.12-1.39)   <0.001 | 2.31 (1.73-3.08)   <0.001 |
| **Model 2: Two-piecewise Cox** | |  |  |  |
| Inflection point (*K*) | 0.70 | 0.58 | 2.31 | 0.80 |
| SHR ≤ *K* | 0.10 (0.02-0.47)   0.003 | 0.21 (0.03-1.36)   0.101 | 1.55 (1.17-2.07)   0.003 | 0.04 (0.01-0.34)   0.004 |
| SHR > *K* | 3.24 (2.19-4.80)   <0.001 | 2.94 (1.93-4.48)   <0.001 | 1.15 (0.96-1.37)   0.120 | 2.69 (2.02-3.58)   <0.001 |
| *P* for non-linearity | <0.001 | 0.016 | 0.109 | 0.001 |
| **365-Day Mortality** | | | | |
| **Model 1: Linear Cox** | 1.50 (1.13-1.99)   0.005 | 1.97 (1.47-2.64)   <0.001 | 1.20 (1.08-1.32)   <0.001 | 1.90 (1.49-2.42)   <0.001 |
| **Model 2: Two-piecewise Cox** | |  |  |  |
| Inflection point (*K*) | 0.89 | 0.69 | 1.10 | 0.94 |
| SHR ≤ *K* | 0.40 (0.20-0.78)   0.008 | 0.52 (0.20-1.36)   0.182 | 0.60 (0.22-1.62)   0.315 | 0.33 (0.11-1.00)   0.050 |
| SHR > *K* | 2.60 (1.80-3.76)   <0.001 | 2.68 (1.88-3.81)   <0.001 | 1.22 (1.10-1.35)   <0.001 | 2.33 (1.80-3.01)   <0.001 |
| *P* for non-linearity | <0.001 | 0.006 | 0.185 | 0.003 |

Admission SHR was calculated using the first blood glucose value measured within 24 hours of ICU admission. Minimum SHR, maximum SHR, and mean SHR were calculated using the minimum, maximum, and mean glucose values within 24 hours of ICU admission, respectively. *P* for non-linearity was determined by log-likelihood ratio test comparing the two-piecewise linear Cox regression model with the standard linear Cox regression model. Models were adjusted for gender, age, race, weight, myocardial infarction, congestive heart failure, peripheral vascular disease, cerebrovascular disease, chronic pulmonary disease, renal disease, diabetes, cancer, WBC, hemoglobin, platelet, BUN, creatinine, potassium, sodium, INR, SOFA score, mechanical ventilation, vasoactive agent use, CRRT, AF type, and infection source. Abbreviation: CI, confidence interval; HR, hazard ratio; SHR, stress hyperglycemia ratio.

**Table S4. E-value sensitivity analysis for unmeasured residual confounding across threshold segments**

| **Outcome/Model** | **Threshold Segment** | **HR (95% CI)** | **E-value** |
| --- | --- | --- | --- |
| **30-Day Mortality** |  |  |  |
| **Multivariable-adjusted Model** | Inflection point (K = 0.70) | — | — |
|  | SHR≤ 0.70 | 0.10 (0.02–0.47) | 19.49 |
|  | SHR >0.70 | 3.24 (2.19–4.80) | 5.93 |
| **Entropy Balancing Weighted Model** | Inflection point (K = 0.70) | — | — |
|  | SHR≤ 0.70 | 0.07 (0.01–0.33) | 28.06 |
|  | SHR > 0.70 | 3.37 (2.28–4.98) | 6.19 |
|  |  |  |  |
| **365-Day Mortality** |  |  |  |
| **Multivariable-adjusted Model** | Inflection point (K = 0.89) | — | — |
|  | SHR≤ 0.89 | 0.40 (0.20–0.78) | 4.44 |
|  | SHR > 0.89 | 2.60 (1.80–3.76) | 4.64 |
| **Entropy Balancing Weighted Model** | Inflection point ($K = 0.77) | — | — |
|  | SHR≤ 0.77 | 0.19 (0.07–0.51) | 9.97 |
|  | SHR > 0.77 | 2.32 (1.67–3.23) | 4.07 |

**Figure S1. Absolute standardized mean differences before and after entropy balancing weighting.** Covariate balance across SHR quartiles before (red dots) and after (blue dots) weighting. The dashed vertical line indicates an SMD threshold of 0.10. Abbreviations: SMD, standardized mean difference; SHR, stress hyperglycemia ratio.

**Figure S2. Subgroup analysis of the association between SHR and mortality.** Forest plots showing hazard ratios (95% CIs) for 30-day and 365-day mortality across clinical strata (N = 2,297). Multivariable-adjusted model.

**Figure S3. Sensitivity analysis of RCS knot specifications.** RCS curves for 30-day and 365-day mortality using 3 knots and 5 knots.

**Figure S4. Sensitivity analysis using median SHR as reference.** RCS curves using cohort median SHR (0.93) as the reference point.

**Figure S5. Sensitivity analysis in trimmed cohort.** RCS curves for 30-day and 365-day mortality after trimming extreme tail observations (<1st and >99th percentiles).
